# Recovery from Resistance Exercise with or Without Blood Flow Restriction Results in an Early Biphasic Pattern of Change in Albumin Cys34 Oxidation in Untrained Males

**DOI:** 10.3390/antiox14060667

**Published:** 2025-05-30

**Authors:** Zi Xiang Lim, Jackson Peos, Stefan Ostojic, Peter G. Arthur, Paul A. Fournier

**Affiliations:** 1 Exercise Physiology and Biomarkers Laboratory, Yong Loo Lin School of Medicine, National University of Singapore, Singapore 117456, Singapore; 2 Department of Biochemistry, Yong Loo Lin School of Medicine, National University of Singapore, Singapore 117456, Singapore; 3 Healthy Longevity Translational Research Program, Yong Loo Lin School of Medicine, National University of Singapore, Singapore 117456, Singapore; 4 School of Human Sciences, Department of Sport Science, Exercise & Health, The University of Western Australia, Perth 6009, Australia; jackson.peos@research.uwa.edu (J.P.); 20740934@student.uwa.edu.au (S.O.); paul.fournier@uwa.edu.au (P.A.F.); 5 School of Molecular Sciences, The University of Western Australia, Perth 6009, Australia; peter.arthur@uwa.edu.au

**Keywords:** antioxidant, low-intensity resistance exercise combined with blood flow restriction, maleimide labeling, protein thiol oxidation, reactive oxygen and nitrogen species

## Abstract

Background: Oxidative stress contributes to the activation of muscle protein synthesis after high-intensity resistance exercise (HIRE) or low-intensity resistance exercise combined with blood flow restriction (LIBFR), but it is unclear if this oxidative stress response post-exercise is monophasic or multiphasic. We aimed to answer this question using albumin Cys34 oxidation as an oxidative stress marker. Methods: Seven untrained individuals completed HIRE and LIBFR on separate days. Albumin Cys34 oxidation (total and reversibly and irreversibly oxidized fractions), muscle oxygenation, oxygen consumption (${\dot{V}O}_{2}$), lactate, and heart rate (HR) were measured before and up to 5 h post-exercise. Results: Both HIRE and LIBFR induced a biphasic increase in total oxidized albumin Cys34, with a transient peak in irreversibly oxidized albumin Cys34 immediately post-exercise (*p* < 0.001) before a delayed sustained increase in reversibly oxidized albumin Cys34, which peaked at 90–120 min and lasted ≥5 h post-exercise (*p* < 0.05). Muscle oxygenation decreased immediately post-exercise (*p* < 0.001) before rising above baseline (*p* < 0.05). ${\dot{V}O}_{2}$, HR, and blood lactate peaked post-exercise (*p* < 0.001) and returned to baseline within 15–90 min. Irreversibly oxidized albumin Cys34 was positively correlated with lactate and ${\dot{V}O}_{2}$ post-exercise (*p* < 0.001). Conclusion: Here, we show that resistance exercise, with or without blood flow restriction, results in an early biphasic oxidative stress response after exercise.

## 1. Introduction

High-intensity resistance exercise (HIRE) provides an effective means to stimulate both the synthesis of muscle protein and muscle hypertrophy [1,2]. A resistance load exceeding 65% of one repetition maximum (1 RM) combined with multiple repetitions (e.g., eight to ten reps) and sets effectively stimulates muscle growth and activation of mammalian target of rapamycin complex 1 (mTORC1) signaling [3]. Interestingly, similar increases in muscle growth and activation mTORC1 signaling can be achieved through multiple repetitions of low-intensity resistance exercise (e.g., 20% of 1 RM) when paired with blood flow restriction (LIBFR) [3,4,5].

The rise in the concentration of reactive oxygen and nitrogen species (RONS) after a bout of HIRE or LIBFR in humans facilitates the synthesis of muscle protein, in part, by modulating the redox state of regulatory proteins that activate the mTORC1 signal transduction pathway [2,6,7,8,9]. However, no study has examined, under high resolution, the temporal pattern of change in the concentration of RONS in the blood or muscle after resistance exercise with or without blood flow restriction. Indeed, most studies on resistance exercise have measured blood markers of oxidative stress at limited timepoints such as immediately, 15 min, 1 h, or 24 h post-exercise [10,11,12,13]. One notable exception is the study of Nikolaidis and colleagues, which examined the pattern of change in oxidative stress over a four-day period after both muscle-damaging and non-damaging exercise [14]. They observed that muscle-damaging exercise resulted in a biphasic oxidative stress response in the blood, with an early peak in oxidative stress within one hour after exercise and a second peak at 24–48 h [14]. One limitation of that study, however, is that only three time points (0, 1, and 4 h) were selected for the early recovery period, thus raising the issue of whether additional early oxidative stress peaks may have gone undetected. This is an important issue to address because a multiphasic oxidative stress response to resistance exercise could reflect a time-dependent recruitment of different RONS-generation sources (e.g., electron transport chain, xanthine oxidase, NADPH oxidase, neutrophil myeloperoxidase), with each being associated, at different times, with post-exercise protein synthesis, muscle damage, and inflammation.

Frequent blood sampling can pose logistical challenges for studies concerned with the temporal tracking of oxidative stress. For this reason, we recently developed the maleimide polyethyleneglycol (mPEG) assay for the non-invasive tracking of albumin Cys34 thiol oxidation, a highly sensitive oxidative stress marker, using just 10 ul of fingertip blood [15], which is far less than the large volume of blood that was required in earlier assays (>1 mL) [16]. Although most plasma protein thiols are oxidized, albumin Cys34 is only partially oxidized and is the predominant source of plasma thiols (>90%). Upon oxidation, Cys34 can be reversibly oxidized to a sulfenic derivative (SOH), form a reversible disulfide bond (-S-S-) with a low-molecular-weight thiol (e.g., glutathione, cysteine), or, if further oxidized, can be converted to irreversibly oxidized forms, such as a sulfinic (-SO_2_H) or sulfonic (-SO_3_H) acid derivative [17]. The increase in the proportion of reversibly and irreversibly oxidized albumin Cys34 in response to an increase in the concentration of RONS has been used as a highly sensitive marker of oxidative stress [15,18,19,20] and has been reported to be more sensitive to changes in oxidative stress than other markers such as the level of protein carbonyls [13].

Since both HIRE and LIBFR cause oxidative stress and considering the inconclusive evidence for a monophasic pattern of change in oxidative stress early during recovery (0–5 h) from damaging exercise [14], our main aim was to test the hypothesis that resistance exercise, with or without blood flow restriction, causes a monophasic pattern of change in oxidative stress early post-exercise.

## 2. Materials and Methods

### 2.1. Participants

Seven male participants aged 18–35 years who were not involved in resistance training in the previous three months prior to testing were recruited for this study (see Table 1 for descriptive characteristics). On the basis of our earlier findings that exercise at maximal intensity is associated with a large effect size for the change in the proportion of thiol-oxidized albumin [15], using the proportion of thiol-oxidized albumin as our primary outcome variable, we calculated that a sample size of seven participants would provide sufficient statistical power (0.8) for our repeated-measures study design to detect a two-sided effect size above 1.4 with a level of significance of *p* < 0.05. Participants were recruited from the student population at the University of Western Australia and from the general population through flyers posted on social networks. Exclusion criteria included supplementation with antioxidants and any medical condition or musculoskeletal injury that could cause oxidative stress or impair exercise performance. The participants were informed about the study’s requirements and risks and were asked to sign a written informed consent form. The study was approved by the Ethics Committee of the University of Western Australia (project number RA/4/1/7555).

### 2.2. Overview of the Experimental Design

**Figure 1:**
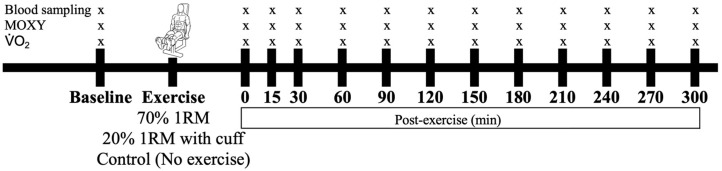
Timeline of data collection before and after the interventions. The participants performed either 70% 1 RM, 20% 1 RM (with cuff), or no exercise (control), with these conditions administered in a randomized order. Symbols: “x” represents the time points at which blood, muscle oxygenation (MOXY), and ${\dot{V}O}_{2}$ were sampled or measured.

All participants were subjected to an HIRE, an LIBFR, or a no-exercise control session on separate days, with exposure to these experimental conditions being randomized and counterbalanced. Before and at time intervals after testing, blood was sampled, the level of muscle oxygenation was recorded, and the proportions of reversibly and irreversibly oxidized albumin cysteine 34 (Cys34) were measured as biomarkers of oxidative stress (Figure 1).

### 2.3. Familiarization Session

Each participant was required to visit the exercise physiology laboratory at the University of Western Australia on four distinct occasions. The first visit involved a familiarization session during which height and weight were recorded, and body composition was analyzed using dual-energy x-ray absorptiometry (DXA, Alpenglow, Sydney, Australia). Also, the maximal bilateral leg extension torque that could not be performed more than once (1 RM) was measured on a standard leg extension machine (Orbit Fitness, Western Australia). To determine the 1 RM of each participant, all participants performed a warm-up with 40 kg resistance before completing a series of single repetition attempts. Each attempt was separated by 2 min of rest, with the resistance increasing by 10 kg at each attempt until concentric failure, defined as the inability to achieve full knee extension.

Following 1 RM determination, the participants underwent a graded exercise test to assess their peak rates of oxygen consumption (${\dot{V}O}_{2}$ peak) with the help of an indirect calorimetry system. The graded test involved stationary cycling starting at an intensity of 50 watts, with the intensity increasing by 30 watts every minute until volitional exhaustion and until reaching a respiratory exchange ratio in excess of 1.10. During this test, the participants breathed through a mouthpiece to collect expired air for gas analysis. Respiratory gases and pulmonary ventilation were measured using a computerized metabolic cart, which included a ventilometer (Universal ventilation meter, VacuMed, Ventura, CA, USA) calibrated according to the manufacturer specifications before each trial. The gas analyzers (Ametek Applied Electrochemistry S-3A/1 and CD-3A, AEI Technologies, Pittsburgh, PA, USA) were also calibrated prior to each testing session using standard gas mixtures with known compositions. The participants were familiarized with all the procedures before leaving the laboratory.

### 2.4. Testing Sessions

At least seven days after the familiarization session, the participants visited the laboratory on three separate occasions, each following an overnight fast. They were instructed to avoid caffeine, medicine, supplements (e.g., antioxidants), and exercise for 24 h prior to each testing session. To ensure consistency in food intake between visits, each participant was given a diary, and dietary analyses were conducted using Foodworks (Xyris Software Pty Ltd., Brisbane, QLD, Australia). All three treatments (LIBFR, HIRE, and control sitting) were administered by adopting a counterbalanced study design, with no less than one week between consecutive visits. Each participant arrived between 6 and 8 AM for every visit to the laboratory and was fitted with a blood pressure cuff around both thighs, a near infrared spectroscopy probe to measure the level of muscle oxygenation, and a heart rate monitor (see below for more details). Finally, the respiratory gases of the participants were collected before exercise and at various time intervals afterwards to measure their rates of oxygen consumption (${\dot{V}O}_{2}$). ${\dot{V}O}_{2}$ recovery kinetics were calculated by subtracting ${\dot{V}O}_{2}$ at baseline from that during the first 5 min of recovery after exercise, followed by summating the area under curve. 

During one of the visits, the participants underwent the LIBFR protocol. A pressure cuff (Joven Technology, VC 12, 12 cm, Winnipeg, MB, Canada) was placed around the most proximal portion of both thighs and inflated to 200 mmHg, as per the protocol of Gundermann and colleagues [9]. The participants then performed four sets of bilateral leg extension, from 90° of knee flexion to full extension (180°), on a standard leg extension machine (Orbit Fitness, Western Australia) as described by Gundermann and colleagues [9]. Each set consisted of 30, 15, and 15 repetitions and 15 repetitions at 20% 1 RM, with each repetition involving 1.5 s of concentric contraction and 1.5 s of eccentric contraction, guided by a metronome, with 30 s of rest between consecutive sets. On a separate occasion, the participants were subjected to the HIRE protocol. To this end, the pressure cuffs were set at 0 mmHg, and the participants performed 8 sets of 10 repetitions of bilateral leg extensions at 70% 1 RM, with each repetition involving 1.5 s of concentric contraction and 1.5 s of eccentric contraction and a three-minute break between consecutive sets [2]. These two protocols of resistance exercise (LIBFR and HIRE) were chosen because they have been reported to result in a similar activation of both mTORC1 and protein synthesis [2]. Finally, the control sitting trial was completed on another visit, during which no exercise was performed and the participants sat calmly with 0 mmHg applied by the pressure cuffs.

Prior to exercise or passive sitting, as well as at 0, 15, 30, 60, 90, 120, 150, 180, 210, 240, 270, and 300 min after exercise or passive sitting, one hand of each participant was placed in a 50 °C hot box (HotRox, Buntingford, UK) for 5 min to arterialize the capillary blood composition. Capillary blood was sampled from the fingertip to measure the blood lactate concentration and the proportion of oxidized albumin Cys34. Respiratory gases were also collected using the indirect calorimetry system described above. Each participant was asked to rate their perceived exertion using a BORG scale [21] at the end of each exercise session.

### 2.5. Measurement of Muscle Oxygenation and Heart Rate

The participants were fitted with a near-infrared spectroscopy (NIRS) probe (MOXY NIRS monitors, Hutchinson, MN, USA) that was placed on the belly of the *vastus lateralis* muscle and positioned midway between the lateral epicondyle and greater trochanter of the femur [22] to assess relative muscle oxygenation levels before exercise, during exercise, and up to five hours post-exercise. The device measured muscle oxygen saturation (SmO_2_) every two seconds and has been previously validated in detail [23]. Additionally, a heart rate monitor (Polar FT, Kempele, Finland) was also fitted to measure heart rate before, during, and after exercise.

### 2.6. Blood Collection and Analyses

The blood that was taken to measure the concentration of lactate was placed in heparinized 35 µL capillary tubes, and this analyte was assayed using a blood gas analyzer (ABL^TM^ 700, Radiometer, Copenhagen, Denmark). Another drop of blood was collected to measure the oxidation state of plasma albumin Cys34 using the mPEG assay described by Lim and colleagues [15]. Briefly, blood was collected in K_3_EDTA tubes (Minicollect tubes K_3_EDTA; Greiner Bio-One, Kremsmünster, Austria). Nine parts of this blood sample was immediately diluted with one part of a trapping solution composed of 62.5 mM methoxypolyethylene glycol-maleimide–5000 Da (Malpeg; Jenkem Technologies, Beijing, China) and 40 mM imidazole (Sigma, Melbourne, Australia), pH 7.4, diluted in DDI water. The mixture was then vortexed briefly before being centrifuged at 3000× *g* for 10 min to separate the plasma (Eppendorf, New York, NY, USA). The plasma obtained was kept at room temperature for another 20 min to allow the Malpeg to react fully with all of the plasma-reduced thiols. Then, the treated plasma samples were frozen in liquid nitrogen and stored at −80 °C until analyzed, as briefly described in the following paragraph.

The plasma samples that had been treated with Malpeg were thawed and analyzed using the mPEG assay [16]. Protein separation, gel processing, membrane processing, and visualization of albumin were performed as described previously [16]. The results for the reversibly oxidized albumin, irreversibly oxidized albumin, and total (irreversible plus irreversible) oxidized albumin were expressed as ratiometric percentages (%) as described previously [15]. One advantage of expressing oxidative stress this way is that the results obtained were not affected by any shift in plasma volume. The inter assay coefficient of variation (CV) for albumin Cys34 is 4.4% (*n* = 16).

### 2.7. Statistical Analyses

The statistical analyses were achieved with the help of the Statistical Package for the Social Sciences for Windows (version 23; SPSS Inc., Chicago, IL, USA). Unless otherwise stated, the data are shown as the mean ± SEM, with statistical significance set at *p* < 0.05. The data that were collected for total oxidized, reversibly oxidized, and irreversibly oxidized albumin, as well as the other variables, were analyzed with the help of a two-way ANOVA with repeated measures (time × intervention). A Fisher LSD test was adopted to uncover differences over time between and within treatments. If Mauchly’s sphericity test was violated (*p* < 0.05), Huynh–Felt correction was adopted. Effect sizes (partial eta squared (η²) and Cohen’s *d*) were calculated, where η^2^ values of 0.01, 0.06, and 0.14 indicate small, medium, and large effects, respectively, and Cohen’s *d* values of 0.2, 0.5, and 0.8 represent small, medium, and large effects [24]. A stepwise multivariate linear regression was used to measure the correlations between several variables (e.g., lactate, muscle oxygenation) and reversibly oxidized and irreversibly oxidized albumin Cys34.

## 3. Results

### 3.1. Matching of Food Intake Prior to Testing

There was no significant difference in total energy and macronutrient intake before the trials, as shown in the self-reported food diaries (*p* > 0.050; Table 2). The participants were not taking any antioxidant or vitamin supplements.

### 3.2. Responses of the Proportions of Total, Reversibly, and Irreversibly Oxidized Albumin Cys34 to HIRE and LIBFR Resistance Exercise

**Figure 2:**
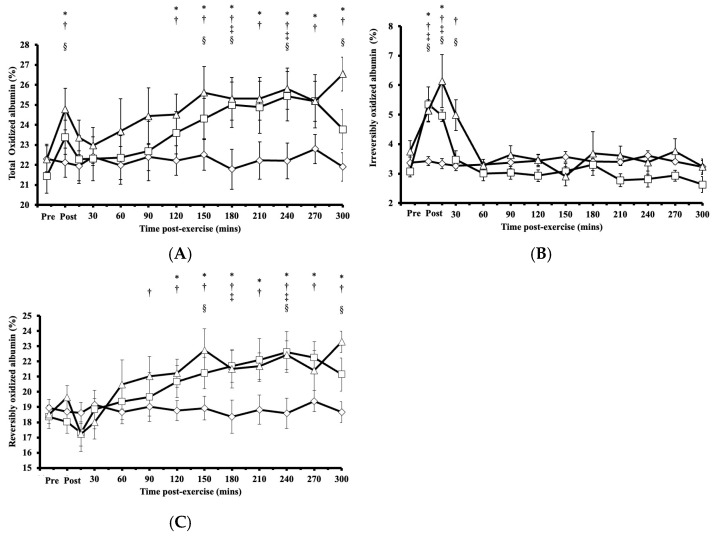
(**A**) Proportion of total oxidized albumin Cys34 levels; (**B**) proportion of irreversibly oxidized albumin Cys34 levels; (**C**) proportion of reversibly oxidized albumin Cys34 in response to the control 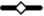, HIRE 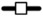, and LIBFR 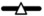 exercise trials. Data are shown as the mean ± SE (*n* = 7). Symbols: *—significant difference from baseline in HIRE trial; †—significant difference from baseline in LIBFR trial; ‡—significant difference between HIRE and control trials; §—significant difference between LIBFR and control trials. Statistical significance set at *p* < 0.05.

Following HIRE or LIBFR, the proportion of total oxidized albumin Cys34 exhibited significant main effects on intervention (*p* < 0.001, η^2^ = 0.272) and time (*p* = 0.003, η^2^ = 0.673), with a biphasic increase compared to baseline and an initial peak immediately at the onset of recovery (*p* < 0.050, Cohen’s *d* = 0.682; Figure 2A). Within 15 min post-exercise, this proportion of total oxidized albumin Cys34 returned to baseline (*p* > 0.050) before rising again at 120 min and then remaining elevated for up to 300 min after exercise (*p* < 0.050, Cohen’s *d* = 0.622–0.959; Figure 2A). No significant difference was observed in the proportion of total oxidized albumin Cys34 between HIRE and LIBFR (*p* = 0.870; Figure 2A), and the proportion of total oxidized albumin did not change during the control sitting condition (*p* > 0.050).

For irreversibly oxidized albumin, there were significant main effects on intervention (*p* < 0.001, η^2^ = 0.456), time (*p* = 0.004, η^2^ = 0.708), and their interaction (intervention × time; *p* = 0.029, η^2^ = 0.789). Following HIRE or LIBFR, there was a significant increase from baseline in the proportion of irreversibly oxidized albumin Cys34, reaching its peak at 0 and 15 min post-exercise (*p* < 0.001, Cohen’s *d* = 0.611–1.272; Figure 2B). Within 30 min post-exercise, the proportion of irreversibly oxidized albumin Cys34 returned to baseline and remained unchanged thereafter (*p* > 0.050). No significant difference was found in the proportion of irreversibly oxidized albumin Cys34 between HIRE and LIBFR (*p* = 0.370; Figure 2B), and the proportion of irreversibly oxidized albumin did not change during the control sitting condition (*p* > 0.050).

For reversibly oxidized albumin, there were significant main effects on intervention (*p* < 0.001, η^2^ = 0.294), time (*p* < 0.001, η^2^ = 0.767), and their interaction (intervention × time; *p* = 0.023, η^2^ = 0.795). The proportion of reversibly oxidized albumin Cys34 increased significantly above baseline at 90 min following LIBFR (*p* = 0.040) and 120 min following HIRE (*p* = 0.048; Figure 2C). These elevated levels persisted for up to five hours post-exercise in both trials (*p* < 0.050, Cohen’s *dd* = 0.732–1.086). There was no significant difference in the proportion of reversibly oxidized albumin Cys34 between HIRE and LIBFR (*p* = 0.600; Figure 2C), and no changes were observed in the proportion of reversibly oxidized albumin during the control sitting condition (*p* > 0.050).

### 3.3. Muscle Oxygenation Level and ${\dot{V}O}_{2}$ Post-Exercise Responses to HIRE and LIBFR Resistance Exercise

**Figure 3:**
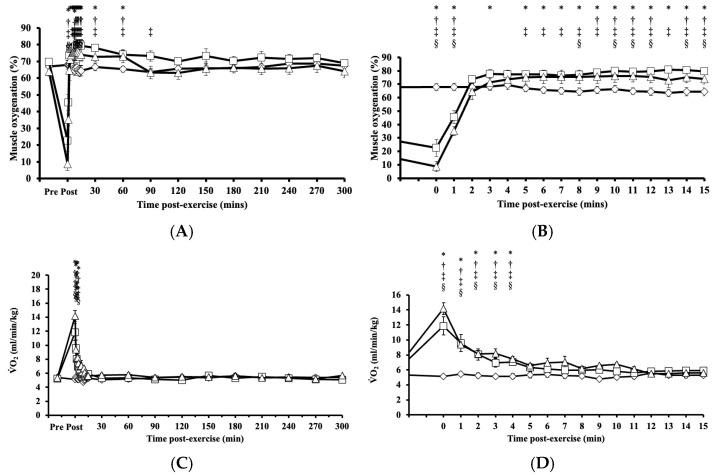
Tissue oxygenation up to 300 min (**A**) with a zoomed-in view of the first 15 min (**B**) post-exercise and the rate of oxygen consumption up to 300 min (**C**) with a zoomed-in view of the first 15 min (**D**) post-exercise in response to the control 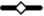, HIRE 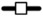, and LIBFR 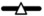 exercise trials. Data are displayed as the mean ± SE (*n* = 7). Symbols: *—significant difference from baseline in HIRE trial; †—significant difference from baseline in LIBFR trial; ‡—significant difference between HIRE and control trials; §—significant difference between LIBFR and control trials. Statistical significance set at *p* < 0.05.

Immediately after HIRE and LIBFR, muscle oxygenation exhibited significant main effects on intervention (*p* = 0.005, η^2^ = 0.223), time (*p* < 0.001, η^2^ = 0.795), and their interaction (intervention x time; *p* < 0.001, η^2^ = 0.691), where the level of muscle oxygenation fell significantly below baseline (*p* < 0.001; Figure 3A,B), then increased above baseline (*p* < 0.050; Figure 3A,B), and remained elevated for 30 to 60 min after exercise before returning to baseline (*p* > 0.050; Figure 3A,B). No significant changes were observed in muscle oxygenation levels between the HIRE and LIBFR trials (*p* = 0.99; Figure 3A,B), and the level of muscle oxygenation remained unchanged during the control sitting trial (*p* = 0.847; Figure 3A,B).

Immediately after HIRE and LIBFR and up to 5 min post-exercise, ${\dot{V}O}_{2}$ remained significantly elevated compared to baseline (*p* < 0.001; Figure 3C,D). No significant difference was observed in ${\dot{V}O}_{2}$ between the HIRE and LIBFR trials (*p* = 0.30; Figure 3C,D), and ${\dot{V}O}_{2}$ remain unchanged during the control sitting trial (*p* > 0.05; Figure 3C,D). Also, ${\dot{V}O}_{2}$ recovery kinetics during HIRE did not differ from ${\dot{V}O}_{2}$ recovery kinetics during LIBFR (16.55 ± 2.33 mL/kg vs. 15.06 ± 1.57 mL/kg, *p* = 0.608), but both were higher than baseline values (*p* < 0.001 and *p* < 0.001, respectively).

### 3.4. Lactate, Heart Rate, and Rate of Perceived Exertion Responses to HIRE and LIBFR

**Figure 4:**
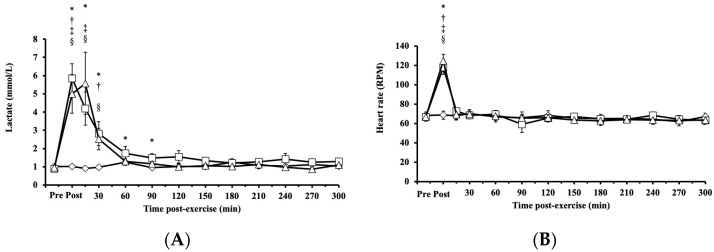
The responses of (**A**) blood lactate and (**B**) heart rate to the control 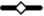, HIRE 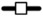, and LIBFR 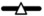 exercise trials. Data are displayed as the mean ± SE (*n* = 7). Symbols: *—significant difference from baseline in HIRE trial; †—significant difference from baseline in LIBFR trial; ‡—significant difference between HIRE and control trials; §—significant difference between LIBFR and control trials. Statistical significance set at *p* < 0.05.

Blood lactate concentrations exhibited significant main effects on intervention (*p* < 0.001, η^2^ = 0.339), time (*p* < 0.001, η^2^ = 0.666), and their interaction (intervention × time, *p* < 0.001; η^2^ = 0.589) and increased significantly after both HIRE and LIBFR, returning to baseline within 30 and 90 min, respectively (*p* < 0.001; Figure 4A). No significant difference was found between HIRE and LIBFR in the concentration of blood lactate (*p* = 0.202; Figure 4A), and the concentration of blood lactate remained unchanged during the control sitting trial (*p* = 0.350; Figure 4A).

Heart rate increased significantly post-HIRE and post-LIBFR (*p* < 0.001; Figure 4B) compared to baseline and returned to baseline within 15 min (*p* > 0.05; Figure 4B). There was no significant difference between HIRE and LIBFR (*p* = 0.98; Figure 4B), and heart rate remained unchanged during the control sitting trial (*p* > 0.05; Figure 4B).

Rate of perceived exertion scores were significantly higher in response to LIBFR and HIRE (19 ± 1; 17 ± 2; *p* < 0.001) than baseline scores, with no significant difference between trials (*p* = 0.12).

### 3.5. Multiple Regression and Correlation Analysis of Cys34

The proportion of irreversibly oxidized albumin Cys34 within the first 90 min of recovery was positively correlated with lactate concentration (*r* = 0.56, *p* < 0.001), heart rate (*r* = 0.41, *p* < 0.001), and ${\dot{V}O}_{2}$ post-exercise (*r* = 0.35, *p* < 0.001; Table 3). There was an inverse correlation between the proportion of reversibly oxidized albumin Cys34 and the concentration of lactate throughout recovery (*r* =−0.128, *p* < 0.001; Table 3). Multiple regression analysis revealed that the concentration of lactate predicted ~30.9% of the proportion of irreversibly oxidized albumin but not reversibly oxidized albumin Cys34 (R^2^ = 0.31, *p* < 0.001; Table 4).

## 4. Discussion

Our primary aim was to test the hypothesis that resistance exercise, with or without blood flow restriction, induces a monophasic pattern of change in oxidative stress shortly after exercise in untrained males. Contrary to expectations, we found that both HIRE and LIBFR resulted in a comparable biphasic, rather than monophasic, change in the proportion of total oxidized albumin Cys34. This response was characterized by an early transient increase in the proportion of irreversibly oxidized albumin Cys34, which returned to baseline within 30 min after exercise. This transient increase in the proportion of irreversibly oxidized albumin Cys34 preceded a delayed, sustained increase in the proportion of reversibly oxidized albumin Cys34, which remained high for at least five hours. Moreover, no significant difference in the proportion of oxidized albumin Cys34 was observed at any time point between the two exercise trials.

Our results are consistent with numerous other studies that have found increases in the concentrations of plasma markers of oxidative stress after resistance exercise [13,25,26]. However, our findings are unique as they show, for the first time, an early biphasic pattern of change in oxidative stress post-resistance exercise. It is noteworthy that most past studies on resistance exercise have measured oxidative stress markers only immediately and/or 15 min after exercise [13,25] or immediately after exercise, one hour later, and/or 24 h after exercise [10,11,12]. This is a limitation because the synthesis of protein and activation of the intramuscular signaling proteins can take up to 3 h to peak after a bout of resistance exercise [2,6,7]. Interestingly, if we had chosen to measure the oxidation state of albumin Cys34 only at the same time points as those chosen in the aforementioned studies, we would not have uncovered the biphasic response of the type shown in Figure 2, thus highlighting the importance of including several sampling time points post-exercise for the type of study described here.

Of note, Nikolaidis and colleagues [14] were the first to show that some types of exercise can result in a biphasic oxidative stress response. Using an exercise modality that differed from those tested here, they found that muscle-damaging exercise resulted in a biphasic oxidative stress response, with a peak in oxidative stress within one hour after exercise and a second peak at 24–48 h after exercise [14]. Since our data collection extended for a period of only 5 h, the findings of Nikolaidis and colleagues [14] raise the intriguing possibility that had our data collection period extended for 48 h or more after exercise, maybe, a third peak in oxidative stress would have been uncovered. This would suggest that resistance exercise paired or not with blood flow restriction may result in a triphasic rather than a biphasic oxidative stress response. Whether this is the case warrants further investigation.

Several intramuscular mechanisms may account for the early post-exercise increase in the proportion of irreversibly oxidized albumin Cys34 [25]. Acute activation of the enzymes/proteins that produce RONS in skeletal muscles, including NAD(P)H oxidase, the electron transport chain, and xanthine oxidase, may be involved [27]. Also, the transient fall in muscle oxygenation during resistance exercise and then rapid reoxygenation [4,28] could lead to increased RONS production [29,30], resulting in the rapid increase in the proportion of irreversibly oxidized albumin Cys34 observed in this study. This latter interpretation is supported by previous studies showing increased ROS generation in response to early ischemia/reperfusion following exercise [31,32], where hypoxia, followed by reperfusion, caused an elevation in the concentration of RONS, possibly due to xanthine oxidase activation [33]. Of note, the lack of an early peak in the proportion of reversibly oxidized albumin Cys34, in contrast to the early peak in the proportion of irreversibly oxidized albumin Cys34, may be explained on the basis that the rate of production of reversibly oxidized albumin Cys34 early after exercise may have been matched by an equivalent rate of conversion of reversibly oxidized albumin Cys34 to its irreversibly oxidized form at the onset of recovery. This would result in no net increase in the proportion of reversibly oxidized albumin Cys34. However, whether this mechanism is involved remains to be determined.

The sustained and delayed increase in the proportion of reversibly oxidized albumin Cys34 post-exercise suggests ongoing intramuscular RONS production. This interpretation aligns with previous findings that peak protein synthesis and activation of intramuscular signaling proteins occur approximately three hours after LIBFR and HIRE [2,6,7]. The delayed rise in reversibly oxidized albumin Cys34, as well as the biphasic pattern of change in the proportion of oxidized albumin Cys34. could also be mediated, at least in part, by the activation of neutrophils since exercise-induced neutrophil translocation to muscles increases RONS production [34,35]. Previous studies have reported that resistance exercise causes biphasic neutrophil activation, peaking early post-exercise and returning to baseline within 30 min of recovery, followed by an increase 2 h later [36,37]. This biphasic pattern of change, which has also been observed in leukocytes [37], suggests the presence of musculoskeletal inflammation. In support of this interpretation, plasma thiols and inflammatory markers have been reported to rise immediately post-exercise before returning to baseline levels, with hypoxia driving neutrophil degranulation and RONS production [38,39].

Our findings that HIRE and LIBFR cause similar patterns of change in the proportion of oxidized albumin Cys34, ${\dot{V}O}_{2}$ post-exercise, tissue oxygenation, heart rate, and lactate suggest that these exercise protocols resulted in comparable oxidative and metabolic stress. This interpretation aligns with protocols resulting in similar activation of intramuscular signaling and muscle protein synthesis [2,6]. Further investigation is required to determine the extent to which the changes in the oxidation state of albumin Cys34 in response to resistance exercise paired or not with blood flow restriction is attributed to neutrophil activation or alterations in intramuscular RONS generation. These changes could, in turn activate mTORC1 signaling, which is involved in muscle protein synthesis.

This study is not without its limitations. Our participants consisted of young untrained males, which restricts our capacity to extrapolate our findings to other populations, such as trained individuals, older individuals, and females. Another limitation is that the antioxidant and vitamin contents of our participants’ diets were not measured. Finally, no attempt was made to identify the RONS-producing and RONS-degrading pathways responsible not only for the biphasic response of the oxidative stress to resistance exercise with or without blood flow restriction but also for the marked difference in the responses of reversibly oxidized and irreversibly oxidized albumin.

## 5. Conclusions

In conclusion, this study is the first to report that resistance exercise, with or without blood flow restriction, leads to an early post-exercise biphasic pattern of increase in oxidative stress in untrained males. Under our experimental conditions, both HIRE and LIBFR induced an early transient post-exercise peak in the proportion of irreversibly oxidized albumin Cys34 that precedes a sustained rise in the proportion of reversibly oxidized albumin Cys34. Also, the proportion of irreversibly oxidized albumin correlated with lactate levels during early recovery, suggesting a link between oxidative stress and metabolic stress. Further research is required to elucidate the mechanisms whereby resistance exercise affects the proportion of reversibly and irreversibly oxidized albumin Cys34.

## Figures and Tables

**Table 1:** Descriptive characteristics of the participants.

Participants’ Physical Characteristics	
Age (years)	23.9 ± 0.9
Weight (kg)	71.4 ± 4.2
Height (m)	1.82 ± 0.04
BMI (kg/m^2^)	21.6 ± 0.9
${\dot{V}O}_{2}$ peak (mL/kg/min)	45.6 ± 1.9
Leg extension 1 RM (kg)	102.1 ± 2.6
20% 1 RM (kg)	20.4 ± 0.5
70% 1 RM (kg)	71.5 ± 1.9
Mid-thigh skinfold (mm)	10.4 ± 1.3
Body fat mass (%)	20.2 ± 2.1
Fat mass (kg)	14.0 ± 1.9
Lean mass (kg)	54.8 ± 3.1

Values are the mean ± SE.

**Table 2:** Dietary intake prior to each testing session.

Diet Prior to Testing	CON	HIRE	LIBFR
Total energy intake (kJ/day)	7749 ± 685	7607 ± 685	7028 ± 390
Fat (g/day)	67 ± 16	59 ± 13	53 ± 13
Fat (% energy intake)	33 ± 4	30 ± 3	29 ± 3
Carbohydrates (g/day)	201 ± 21	207 ± 20	197 ± 17
Carbohydrates (% energy intake)	45 ± 2	47 ± 2	48 ± 2
Protein (g/day)	102 ± 13	103 ± 13	96 ± 13
Protein (% energy intake)	22 ± 1	23 ± 1	23 ± 1

Values are the mean ± SE (*n* = 7).

**Table 3:** Correlation analyses relating the oxidation state of albumin Cys34 to tissue oxygenation, ${\dot{V}O}_{2}$ post-exercise, heart rate, and lactate levels.

		Tissue Oxygenation	${\dot{V}O}_{2}$ Post-Exercise	Heart Rate	Lactate
Irreversibly	Pearson correlation	−0.317 *	0.353 *	0.411 *	0.555 *
oxidized					
albumin	N	260	256	260	247
Reversibly	Pearson correlation	0.076	0.004	0.057	−0.128 *
oxidized					
albumin	N	260	256	260	247

* Correlation is significant at the 0.05 level (2-tailed).

**Table 4:** Multiple regression analyses of irreversibly oxidized albumin and other associated variables.

Variables	Regression Coefficient	Standard Error	Standardized Regression Coefficient	*p*
(Constant)	3.528	0.926		<0.001 *
Lactate	0.313	0.042	0.492	<0.001 *
${\dot{V}O}_{2}$ post-exercise	−0.067	0.05	−0.12	0.180
Tissue oxygenation	−0.012	0.006	−0.147	0.055
Heart rate	0.009	0.005	0.138	0.083
Glucose	0.042	0.098	0.023	0.666

Dependent variable: Irreversibly oxidized albumin. R^2^ for entire model = 0.341. * R^2^ for model lactate only = 0.309.

## Data Availability

The original contributions presented in this study are included in the article; further inquiries can be directed to the corresponding author.

## Abbreviations

The following abbreviations are used in this manuscript:

-SO_2_H	sulfinic acids
-SO_3_H	sulfonic acids
-S-S-	reversible disulfide bond
1 RM	one repetition maximum
albumin Cys34	albumin cysteine 34
HIRE	high intensity resistance exercise
LIBFR	low intensity resistance exercise combined with blood flow restriction
Malpeg	methoxypolyethylene glycol-maleimide
mTORC1	mammalian target of rapamycin complex 1
RONS	reactive oxygen and nitrogen species
SmO_2_	muscle oxygen saturation
${\dot{V}O}_{2}$	rate of oxygen consumption
